# Integrated Proteomic and Transcriptomic-Based Approaches to Identifying Signature Biomarkers and Pathways for Elucidation of Daoy and UW228 Subtypes

**DOI:** 10.3390/proteomes5010005

**Published:** 2017-02-03

**Authors:** Roger Higdon, Jessie Kala, Devan Wilkins, Julia Fangfei Yan, Manveen K. Sethi, Liang Lin, Siqi Liu, Elizabeth Montague, Imre Janko, John Choiniere, Natali Kolker, William S. Hancock, Eugene Kolker, Susan Fanayan

**Affiliations:** 1 Bioinformatics and High-Throughput Analysis Laboratory, Seattle Children’s Research Institute, Seattle, WA 98101, USA; roghig@yahoo.com (R.H.); elizabeth.montague@seattlechildrens.org (E.M.); john.choiniere@gmail.com (J.C.); egnklkr@gmail.com (E.K.); 2 Data and Analytics, Seattle Children’s Hospital, Seattle, WA 98101, USA; imrepnw@live.com (I.J.); ncherryus@gmail.com (N.K.); 3 Data-Enabled Life Sciences Alliance (DELSA), Seattle, WA 98101, USA; 4 High-Throughput Analysis Core, Seattle Children’s Research Institute, Seattle, WA 98101, USA; 5 Department of Chemistry and Biological Sciences, Macquarie University, Sydney 2109, Australia; jessiekala7@yahoo.com (J.K.); wilkins.devan@gmail.com (D.W.); manveen.sethi@students.mq.edu.au (M.K.S.); 6 Department of Chemistry and Chemical Biology, Northeastern University, Boston, MA 02115, USA; fangfei3.yan@gmail.com (J.F.Y.); wi.hancock@neu.edu (W.S.H.); 7 Beijing Genomics Institute, Shenzhen 518083, Guangdong, China; linl@genomics.org.cn (L.L.); siqiliu@genomics.org.cn (S.L.); 8 Chinese Academy of Sciences, Beijing 100101, China; 9 Department of Biomedical Sciences, Macquarie University, Sydney NSW 2109, Australia; 10 Departments of Biomedical Informatics and Medical Education and Pediatrics, University of Washington School of Medicine, Seattle, WA 98195, USA

**Keywords:** medulloblastoma, Daoy, UW228, SHH subtype, WNT subtype, transcriptome, proteome

## Abstract

Medulloblastoma (MB) is the most common malignant pediatric brain tumor. Patient survival has remained largely the same for the past 20 years, with therapies causing significant health, cognitive, behavioral and developmental complications for those who survive the tumor. In this study, we profiled the total transcriptome and proteome of two established MB cell lines, Daoy and UW228, using high-throughput RNA sequencing (RNA-Seq) and label-free nano-LC-MS/MS-based quantitative proteomics, coupled with advanced pathway analysis. While Daoy has been suggested to belong to the sonic hedgehog (SHH) subtype, the exact UW228 subtype is not yet clearly established. Thus, a goal of this study was to identify protein markers and pathways that would help elucidate their subtype classification. A number of differentially expressed genes and proteins, including a number of adhesion, cytoskeletal and signaling molecules, were observed between the two cell lines. While several cancer-associated genes/proteins exhibited similar expression across the two cell lines, upregulation of a number of signature proteins and enrichment of key components of SHH and WNT signaling pathways were uniquely observed in Daoy and UW228, respectively. The novel information on differentially expressed genes/proteins and enriched pathways provide insights into the biology of MB, which could help elucidate their subtype classification.

## 1. Introduction

Medulloblastoma (MB) is the most common malignant brain tumor, with a greater incidence (10–20 times) in young people under 20 years of age [1,2]. Although the survival rate for medulloblastoma patients has improved significantly with the use of combined treatments consisting of surgery, radiation and chemotherapy, the intense and harsh nature of such treatments often leave patients with lifelong disabilities, in particular young patients [3,4]. Novel targeted therapies are needed to alleviate the devastating side effects that result from the toxic nature of the currently available treatment options. Better characterization of each MB subtype could lead to further insights into the biological pathways, which may lead to better treatment strategies tailored to individual tumors and improved outcome. 

The latest classification for medulloblastoma by the World Health Organization (WHO), based primarily on morphology (histopathology), recognized five histological variants of the disease: classic medulloblastoma, desmoplastic/nodular (D/N), medulloblastoma with extensive nodularity (MBEN), anaplastic medulloblastoma and large cell medulloblastoma [5]. However, this classification, based on histology alone, fails to recognize the heterogeneity of the disease. Recent advances in high-throughput genomic and proteomic technologies have greatly enhanced our understanding of medulloblastoma biology. Several studies, employing transcriptional profiling, have shown that medulloblastoma is not a single disease, but a collection of clinically and molecularly diverse tumor subgroups [6,7,8,9]. Recently, Kool et al., through a meta-analysis of all available molecular and clinical data for medulloblastoma, identified four consistent core molecular subgroups of medulloblastoma across all published datasets with distinct clinical, genetic and prognostic features [10]. These combined efforts resulted in a consensus at a recent conference in Boston in 2010, supporting the classification of medulloblastomas, based on the molecular profiling, into four clinical and molecular subtypes: the WNT subtype, in which the canonical WNT signaling is upregulated; the sonic hedgehog (SHH) subtype, with hallmark activation of the SHH-signaling cascade; and two additional groups called “group 3 (G3)” and “group 4 (G4)” medulloblastoma for which the specific signaling pathways leading to these two variants have not yet been conclusively determined [11].

Each medulloblastoma subgroup is characterized by a specific set of genetic mutations, gene amplifications (oncogenic drivers) or deletions (possible loss of tumor suppressors) [12,13,14]. The WNT and SHH subtypes are based on mutations, deletions or over-expression of genes associated with the WNT and SHH signaling pathways, respectively, while the G3 and G4 subgroups are less specifically defined at the molecular level [12,14,15]. Table 1 provides a brief clinical and molecular description for each medulloblastoma subtype. 

The WNT tumors are thought to be the rarest MB subgroup (accounting for ~10% of MB cases), but the best characterized subtype, mainly due to their favorable prognosis, with a survival rate greater than 90% [11,12]. Unlike other subtypes, genetic aberrations are not frequent in this subtype, with only a few mutations and copy number variations observed. One such aberration is a point mutation on exon 3 of the β-catenin gene (*CTNNB1*), identified in over 75% of the tumors, resulting in overexpression and nuclear localization of β-catenin [16,17]. Other genetic variations observed in this subtype include deletion of one copy of chromosome 6 (monosomy 6, observed in almost all cases) and mutations in the DEAD-box RNA helicase gene (*DDX3X*), targeting almost half of all WNT subtypes, which increases the transactivating capacity of β-catenin, resulting in enhanced cell proliferation. Mutations in *SMARCA4* (26%), *TP53* (16%) and *KMT2D* (12%) genes have also been reported [18,19,20,21]. 

The SHH tumor subtype is initiated by aberrant activation of the sonic hedgehog signaling pathway through either mutation, amplification or deletion of genes encoding components of this pathway. Approximately 25% of SHH tumors carry mutations in the individual components of the SHH pathway, with mutation in patched 1 (*PTCH1*) being the most commonly observed [6,12,22,23]. Other aberrations associated with this subtype include amplifications of GLI family zinc finger 1 or 2 (*GLI1*/*GLI2*) [24] and high levels of *MYCN* expression [9]. 

Groups 3 and 4 are less well defined, with their underlying pathway perturbations yet to be elucidated. Group 3 tumors account for around 25% of all medulloblastomas and are more common in males [11,12]. Prognosis is worst in group 3 patients who often present with metastatic disease [9]. This group is further divided into 3α and 3β subgroups, based on *MYC* expression, with 3α tumors closely associated with *MYC* amplification and a poor prognosis, while 3β subsets do not overexpress *MYC* and exhibit an intermediate prognosis [11,12]. Group 4 is the most common and least defined of all MB subtypes, accounting for about 35%–40% of all MB cases. Patients within this subgroup have an intermediate prognosis, with approximately one third presented with metastatic spread. Common aberrations observed in this subgroup include mutations in *KDM6A* (a histone H3 lys27 demethylase, also known as UTX), amplification of *CDK6* and *MYCN*, loss of chromosome X, isochromosome 17q (in 80% of cases) and deletion of 17p (also observed in group 3) [11,12,25]. 

In this study, we employed a mass spectrometry-based proteomic approach, coupled with RNA-Seq analysis, to generate a detailed protein and RNA database for two established primary medulloblastoma cell lines, namely Daoy and UW228 [26,27]. Despite having been established many years ago and extensively used in medulloblastoma studies, detailed interrogation of their transcriptome and proteome has not yet been performed. Peyrl et al. [28] performed proteomic analysis of Daoy by two-dimensional gel electrophoresis, followed by matrix-assisted laser desorption/ionization and identified more than 200 proteins. Recently, Martelli et al. [29] performed a top-down LC-MS proteomic analysis of Daoy, which identified 53 proteins. Conversely, the only detailed proteomics study for UW228 was reported by Zanini et al. [30], who compared the proteomes of Daoy and UW228, cultured in adherence or as medullospheres, to determine the influence of growth conditions on the modulation of protein expression. Moreover, the exact MB subtype to which Daoy and UW228 belong is not clearly established. In a recent study, Pambid et al. [31] classified Daoy as being SHH subtype. However, another study by Othman et al. [32], in which they investigated subtype specific gene expression in several MB cell lines, found it difficult to classify Daoy. 

We applied proteomic and transcriptomic approaches to identify potential signature proteins and pathways that could distinguish between these two cell lines and evaluate variations in transcript and protein expression and their possible relation to subtype specificity of each cell line. 

## 2. Materials and Methods

### 2.1. Materials

Human primary medulloblastoma cell lines, Daoy and UW228, were kindly donated by Dr Peter Dallas (Telethon Institute for Child Health Research, Subiaco, WA, Australia). RPMI 1640 and DMEM media, human recombinant insulin, glutamine, fetal bovine serums (FBS), molecular weight protein standards and NuPage 4%–12% Bis-Tris Mini gels were purchased from Invitrogen (Carlsbad, CA, USA). Protease inhibitor cocktail tablets (EDTA-free) were purchased from Roche Diagnostics (GmbH, Mannheim, Germany). Bradford dye reagent, Nonidet P40 (NP-40), 4-(2-hydroxyethyl)-1-piperazineethanesulfonic acid (HEPES) were purchased from Sigma-Aldrich (St. Louis, MO, USA).

### 2.2. Cell Culture and Preparation of Total Cell Lysate

Human medulloblastoma cell lines Daoy (from a 4-year old male) and UW228 (from a 9-year old female) [27,28] were grown in DMEM media, supplemented with 10% FBS and l-glutamine (1%) at 37°, 5% CO_2_. Cells were washed 3 times in ice-cold PBS and then collected, using a cell scraper, in lysis buffer containing 10 mM Tris-HCl, pH 7.5, 150 mM NaCl, 1% *v*/*v* Nonidet P-40 and protease inhibitor cocktail (Roche Diagnostics, Dee Why, Australia). The suspended cells were incubated for 30 min and ultra-sonicated (Branson Sonifier 450, VWR, Mississauga, ON, Canada) at intervals of 15 s for a total of 2 min, with 15 s pause between each treatment. This was followed by centrifugation at 17,000× *g* for 1 h. All the above steps were performed at 4 °C. Three biological replicates were performed for each analysis. 

### 2.3. One-Dimensional SDS-PAGE Electrophoresis and Trypsin In-Gel Digestion of Total Cellular Proteins and Analysis by LC-MS/MS 

Samples (70 μg total lysate protein per lane) were loaded onto NuPage 4%–12% Bis-Tris precast gradient mini gel (Invitrogen, San Diego, CA, USA). Electrophoresis conditions were set to 200 V, 125 mA for 60 min. Gels were fixed in 10% (*v*/*v*) acetic acid and 40% (*v*/*v*) methanol and stained overnight with Coomassie Blue (Invitrogen, San Diego, CA, USA), followed by destain with 10% (*v*/*v*) acetic acid until background was clear. Proteins in each slice (10 slices per lane) were reduced, alkylated and subjected to trypsin digestion. 

The tryptic digests were analyzed by liquid chromatography-tandem mass spectrometry, using a Q-Exactive mass spectrometer (Thermo Scientific, Scoresby, VIC, Australia). Peptide mixtures in 0.1% (*v*/*v*) formic acid were loaded onto a C_18_ reversed phase column, packed in-house (2.7 μm (particle size) HaloLink Resins, Promega, column dimensions: 100 mm (length) × 75 μm (ID)). Separation of peptides was performed over a 60 min gradient with the first 50 min of the linear gradient increasing from 0% to 50% in solvent B (0.1% (*v*/*v*) aqueous formic acid/100% (*v*/*v*) acetonitrile) and then to 85% in solvent B for the next 2 min and maintained at 85% for 8 min. The flow rate was constant at 300 nL/min. The Easy-nLC (Thermo Scientific) was connected directly to the nano-ESI source of the Q-Exactive. MS full scans were acquired with resolution of 35,000 in the positive ion mode over *m*/*z* 350–2000 range and an automatic gain control (AGC) target value of 1 × 10^6^. The top 10 most intense precursor ions were then isolated for MS/MS using higher energy collisional dissociation fragmentation at 17,500 resolution with the following settings: collision energy: 30%; AGC target: 2 × 10^5^; isolation window: *m/z* 3.0; and dynamic exclusion enabled. Precursors with unassigned or *Z* = +1 charge states were ignored for MS/MS selection.

### 2.4. Protein Identification and Data Analysis

LC-MS/MS raw data were converted to the mzXML format using the freeware ReAdW.exe program (http://www.ionsource.com/functional_reviews/readw/t2x_update_readw.htm) and processed using Proteome Discoverer platform (version 1.3, Thermo Scientific), interfaced with an in-house Mascot server (Matrix Sciences, version 2.3.0, London, UK), was used for data parsing and protein identification. MS/MS datasets were searched against the human UniProt database (released April 2014, 20266 entries) using the Mascot 2.4 algorithm (Matrix Sciences) after Mascot generic file (mgf) generation according to the following criteria: peptide mass tolerance of ±10 ppm, fragment mass tolerance of 0.1 Da, maximum of 2 missed tryptic cleavage sites. Deamidation (N, Q) and oxidation (M) were allowed as potential variable modifications and carbamidomethylation (C) as a fixed modification. Peptides were considered to be present if peptide false discovery rate (FDR) was less than 1%, based on decoy database matches [33]. Peptide and protein grouping according to Proteome Discoverer’s algorithms were allowed, applying strict maximum parsimony principle. If a peptide had more than 1 protein match, it was mapped to the protein with the most peptide matches. If there was no difference, the first protein listed was chosen as the representative protein. A unique protein with at least two unique proteotypic peptides of ≥9 amino acids, with an FDR < 0.01, was qualified for further analysis. The peptide/protein identifications were further analyzed using various in-house and online functional annotation and bioinformatics tools for protein pathway analysis. All proteomics data presented in this work are available via ProteomeXchange with identifier PXD002659.

### 2.5. RNA Extraction

Total RNA was isolated using Trizol reagent (Sigma-Aldrich, Castle Hill, Australia), according to the manufacturer’s instructions and as described previously [34]. Briefly, 1 mL of Trizol was added per 5–10 × 10^6^ cells, followed by repeated shearing (20×) to lyse the cells using a 23/26G needle and incubation for 5 min at room temperature. Chloroform (200 μL) was added and vigorously mixed, followed by incubation at room temperature. Sample was centrifuged at 12,000× *g* for 15 min at 4 °C and the upper aqueous layer was carefully transferred to a fresh tube and RNA was precipitated with isopropanol. The RNA pellet was collected by centrifugation at 12,000× *g* for 10 min at 4 °C, washed twice with 75% ethanol, air-dried and resuspended in RNase-free water. The concentration of RNA was determined using Nanodrop 2000 spectrophotometer (260/280 nm). RNA samples were stored at −80 °C until further use.

### 2.6. cDNA Library Preparation and RNA Sequencing

RNA preparation and sequencing were performed following the protocols outlined by Nagalakshmi et al. [35]. Briefly, total RNA (7 μg) was subjected to two rounds of hybridization to oligo (dT) beads (Invitrogen, Carlsbad, CA, USA) to enrich mRNA. Ribosomal RNA contamination was evaluated by RNA pico chip, using a BioAnalyzer (Agilent, Santa Clara, CA, USA). The resulting mRNA was used to prepare cDNA libraries, using RNA sequencing sample preparation kit (Illumina, San Diego, CA, USA). Sample1 and Sample2 were sequenced using Illumina Genome Analyzer, followed by Genome Analyzer II, which generated four data sets: S1-R1, S2-R1, and S1-R2, S2-R2, respectively.

RPKM (reads per kilo base per million mapped reads, a representation of absolute abundance of transcripts and a measure of gene expression) was used as a measure of relative transcript abundance. Replicates of individual cell lines were averaged and a threshold of 0.4 RPKM in at least one cell line was applied to retain genes for comparison of gene expression [36]. 

## 3. Results 

### 3.1. Proteomic Analysis of Daoy and UW228

**Figure 1:**
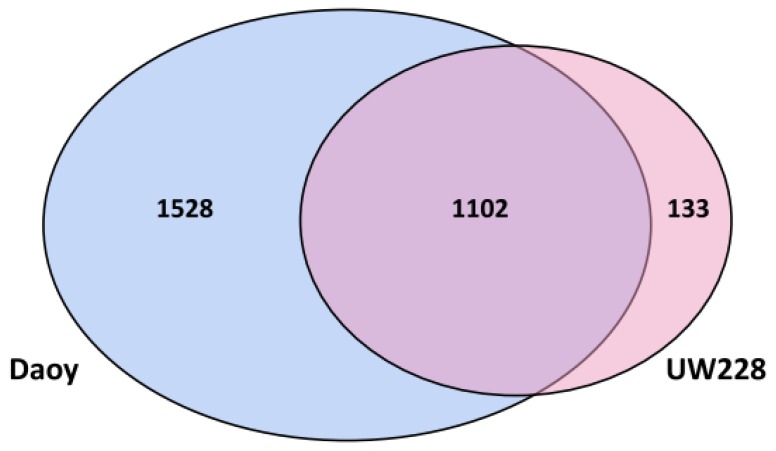
Venn diagram showing common and unique proteins in Daoy and UW228. Shown in the Venn diagram is the distribution of common and unique proteins, identified by 2 or more peptides, in Daoy and UW228. More than half (58%) of the proteins identified in Daoy were not detected in UW228 while only 10% of UW228 proteins were unique.

Proteomic data from Daoy and UW228 cell lines were compared to each other to identify potential signature proteins and pathways, which could help distinguish between these two cell lines. The number of non-redundant proteins, identified with at least two unique peptides, were 2630 for Daoy and 1235 for UW228 (Supplemental Table S1). The complete list of proteins included additional 1543 proteins in Daoy and 1337 proteins in UW228, identified with only one unique peptide, which were excluded from the subsequent analysis (data not shown). Although a large proportion of the proteins identified were common to both cells, 58% of proteins expressed by Daoy (1528 proteins) were not detected in UW228, while UW228 expressed only 133 unique proteins (10% of total proteins), which were not detected in Daoy (Figure 1). Using the analysis approach defined in the methods section, we identified ~800 proteins with differential expression between Daoy and UW228. Of these, at least 150 proteins also exhibited similar expression patterns at the transcript level (i.e., at least 2-fold differentially expressed). Daoy proteome included 358 proteins, which were overexpressed (≥2 fold) relative to UW228 (Supplemental Table S2). Notably, the transcripts for these proteins were also upregulated in Daoy, relative to UW228 (Supplemental Table S2). Conversely, only 27 proteins exhibited higher expression levels (≥2-fold), at both transcript and protein levels, in UW228, compared to Daoy (Supplemental Table S3). Included in the list were proteins involved in cell adhesion (ITGA5, LAMC1, L1CAM, MCAM), migration (AGRN, VCAN, CSPG4), a number of collagen proteins (COL1A2, COL3A1, COL5A2, COL11A1, COL12A1 and COL18A1) as well as some potential tumor suppressors (CRYAB, UTRN, BASP1).

### 3.2. Correlation and Comparison of Proteomics and RNA-Seq Data

The proteomic approach was integrated with the whole-transcriptome shotgun sequencing technology (RNA-Seq), which provides an unbiased and comprehensive profile of the transcriptome. Employing RNA and protein profiling technologies in parallel has been used in recent years to gain a more complete understanding of the cellular systems at both genomic and proteomic levels [37,38,39]. Until recently, it was generally assumed that there was a proportional relationship between mRNA and protein expressions measured from the same sample. However, recent transcriptomic and proteomic studies, from the same cells and under similar conditions, have shown that the correlation between mRNA and protein expressions can be low due to various factors such as post-transcriptional regulation, translation efficiency and variations in protein half-life [40,41,42]. Gene expression data are useful for identifying potential biomarkers and pathways, while proteomics studies are required to validate whether differences in gene expression are truly reflected at the protein level. Therefore, a joint analysis of the transcriptomic and proteomic data can provide useful insights that may not be deciphered from individual analysis of mRNA or protein expression.

Applying a threshold of 0.4 RPKM (reads per kilo base per million mapped reads, a representation of absolute abundance of transcripts and a measure of gene expression) for gene expression in our analysis, approximately 14,470 and 12,810 protein-coding genes were identified for Daoy and UW228, respectively (Supplemental Table S4). The corresponding proteins detected by LC-MS/MS analysis (identified with at least two unique peptides; Supplemental Table S1) were ~18% (for Daoy) and ~10% (for UW228) of the detected transcripts in these cell lines. Interestingly, while the levels of detected transcripts were quite comparable between the two cell lines, almost double the number of proteins were detected in Daoy, compared to UW228.

### 3.3. Analysis of Proteins Differentially Expressed between Daoy and UW228 

The proteome and transcriptome of Daoy and UW228 cell lines were further mined to gain a better understanding of the biological differences between these MB subtypes, including their different growth characteristics. Daoy cells are characteristically more invasive than UW228, with a faster rate of cell division and migration [43]. Table 2 includes a list of selected proteins, some with known cancer association, exhibiting differential expression, at both transcript and protein levels, between the two MB cell lines.

Proteins involved in cell adhesion (POSTN, MCAM, L1CAM and several collagen and integrin proteins), actin-based cytoskeletal organization (LASP1 and several myosin proteins), stress resistance (CRYAB), cell signaling (DAB2, CSPG4), and tumor suppression (BASP1, UTRN) were found to be upregulated at both transcript and protein levels in UW228 cells, relative to Daoy (Table 2 and Supplemental Table S3). Upregulated proteins in Daoy included several proteins involved in signal transduction (EGFR, CRK), cell adhesion (JUP, TGFBI), tumor invasion and metastasis (TNC), stress resistance (HSP90AA1, HSP90AB1) and DNA binding/repair (TOP2A, MSH2 and MSH6). Increased TP53 transcript and protein levels in Daoy (TP53 protein was not detected in UW228) were other notable observations.

Reduced expression of several actin binding proteins (at both transcript and protein levels) in Daoy (compared to UW228) was another interesting observation. Notable among the down-regulated proteins was transgelin (TAGLN). TAGLN is involved in many biological functions, including tumor suppression [44]. It was first characterized by Shield et al. who reported its loss of expression by the RAS oncogene, and as an early event for tumor progression in breast and colon cancers [45]. Suppression of TAGLN expression has subsequently been reported in other cancers [46,47].

**Figure 2:**
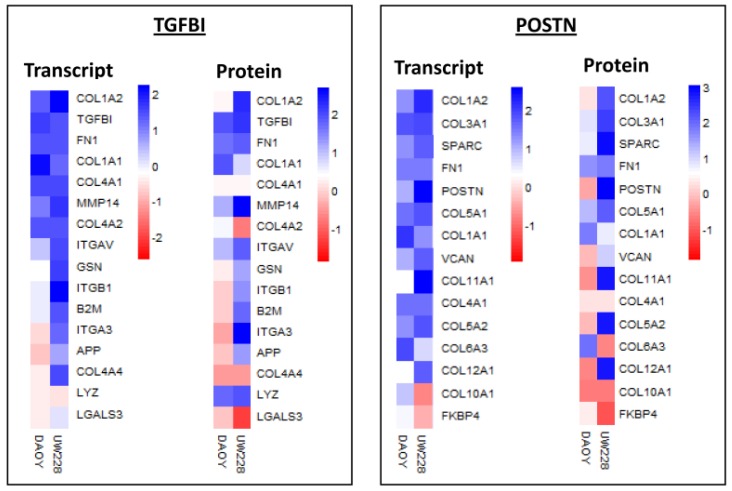
Heat map showing transcript and protein levels for top interacting proteins for TGFBI and POSTN (from STRING) in Daoy and UW228 medulloblastoma cell lines. Blue, white and red squares indicate high, moderate, and low expression, respectively.

Among the proteins with significant differential expression between the two cell lines were TGFBI and POSTN, two extracellular matrix proteins, closely related in structure and function and implicated in tumorigenesis and metastasis [48,49]. TGFBI exhibited the highest expression at transcript level (with relatively high protein level) in Daoy, while the POSTN transcript level was significantly upregulated in UW228. We compared the expression levels of the top interacting proteins for both TGFBI and POSTN (STRING, version 9.0) in both cell lines. Significant upregulation of a number of proteins (including collagens, integrins, fibronectin) was observed in both MB cell lines, but was more apparent in Daoy at both transcript and protein levels (Figure 2).

Interestingly, a number of the amplified transcripts/upregulated proteins (in particular in Daoy) are localized on chromosome 17 (chromosome arms 17p and 17q), which are frequently gained in several malignancies, including medulloblastoma (Table 2 and Supplemental Table S2).

### 3.4. Signaling Pathways Involved in Daoy and UW228

While Daoy and UW228 are routinely used in studies of medulloblastoma, their exact subtype is still poorly understood, with suggestions that Daoy may belong to the SHH subtype. As noted earlier, the SHH subtype is named after the SHH pathway, which is believed to be heavily involved in the pathogenesis of this MB subtype. It is normally initiated by either amplification (oncogenic drivers) or deletion (loss of tumor suppressors) of genes that are components of the SHH pathway. Perturbed signaling in the central developmental signaling pathways such as SHH, wingless (WNT) and the receptor tyrosine kinase ErbB family have in fact been implicated in the formation of MB [50,51,52]. 

**Figure 3:**
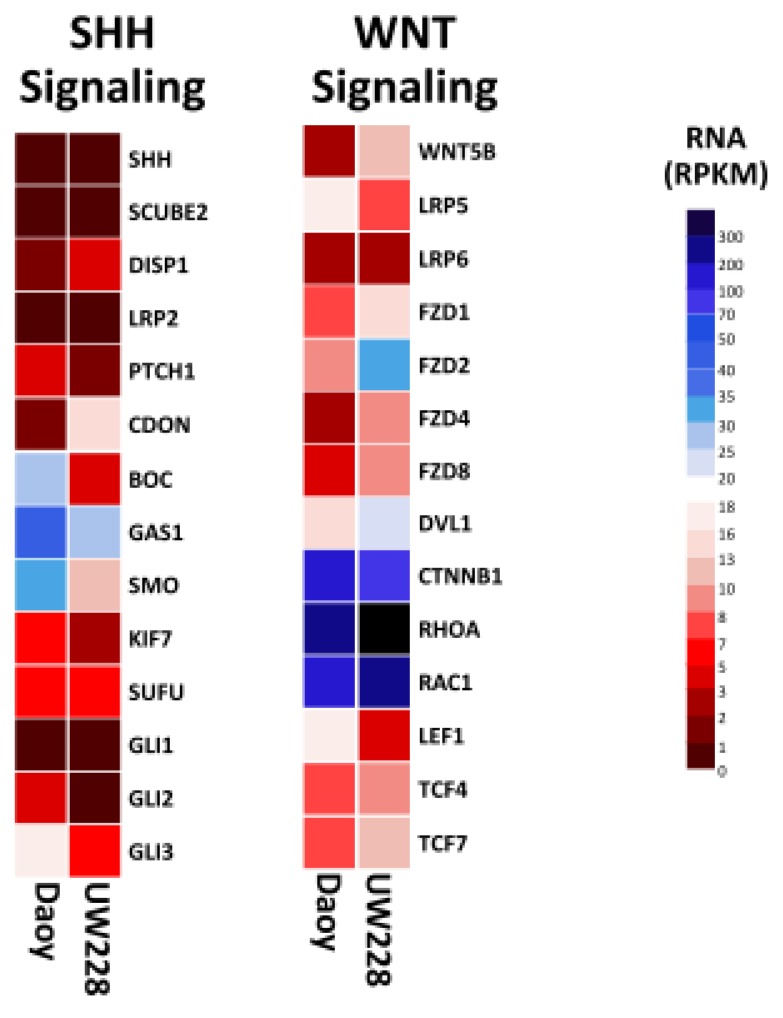
Heat map showing transcript expression of key components for SHH and WNT, signaling pathways in Daoy and UW228 medulloblastoma cell lines. Enrichment of several integral components of WNT signaling pathway were observed in UW228, compared to Daoy. Conversely, increased expression of several elements of the SHH signaling pathway were evident in Daoy, compared to UW228. Blue, white and red squares indicate high, moderate, and low expression, respectively.

In an effort to identify protein markers and pathways that would help elucidate their subtype classification, we mined the proteome and transcriptome of Daoy and UW228 cell lines to examine the expression levels of key components of these pathways. A notable observation was that ≥80% of component genes in both pathways were detected with RPKM values of ≥0.3 in both cell lines. Interestingly, the more invasive Daoy cells exhibited enrichment of the core components of the SHH pathway, compared to UW228 (Figure 3). Higher transcript levels for the GLI family was observed in Daoy, relative to UW228, consistent with other studies of frequent amplification of the *GLI* family in the SHH-subtype medulloblastoma [24]. Conversely, upregulation of several integral components of the WNT signaling pathway was observed in UW228, compared to Daoy, which may suggest a role for this pathway in UW228 biology (Figure 3).

**Figure 4:**
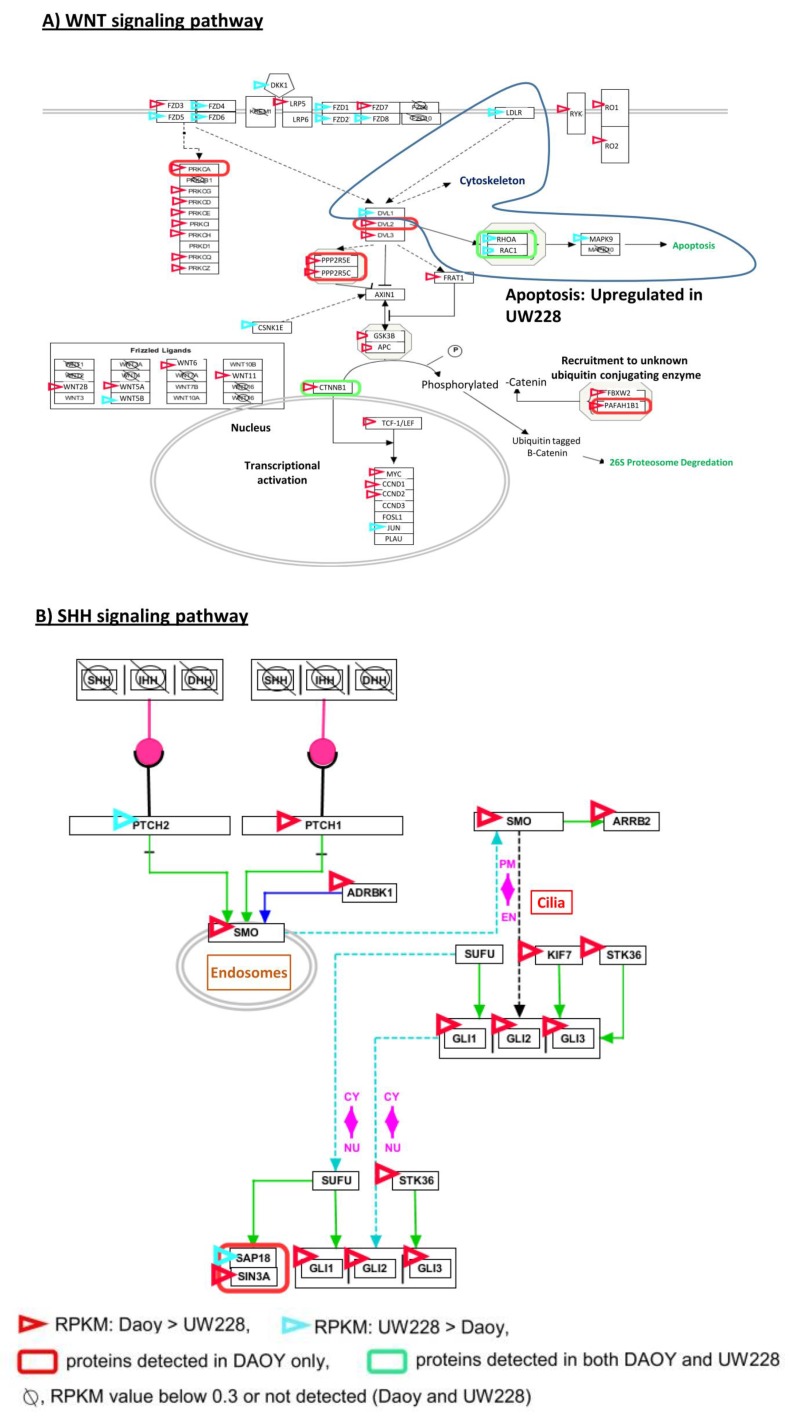
Annotation of WNT (**A**) and SHH (**B**) signaling pathways with transcriptomic and proteomic data from Daoy and UW228. The pathways were derived from http://www.pathvisio.org/.

We next searched for potential sub-pathways within the SHH and WNT signaling pathways in which differentially expressed proteins are involved. Several interesting observations were made. Differential expression of components of different sub-pathways within the WNT signaling pathway, between Daoy and UW228, was a notable observation and consistent with our data presented in Figure 4. The apparent deregulation of the apoptosis sub-pathway (marked with a blue circle), within the WNT pathway, whose component genes were detected exclusively in UW228 (Figure 4A), was an important observation. Within the SHH pathway, upregulation of majority of component genes (marked with red triangles) and detection of some components at protein level (green box) were observed exclusively in DAOY, which, together with data presented in Figure 3, may suggest a role for this pathway in DAOY biology (Figure 4B).

## 4. Discussion and Conclusions

Medulloblastoma is the most common malignant tumor of the primary central nervous system in children, with less frequency of incidence in adults. Prognosis and treatment are similar for both adult and pediatric medulloblastoma. Recent advances in transcriptome and proteome profiling of medulloblastoma have identified it as a heterogeneous malignancy comprised of several subtypes, each with distinct molecular features and clinical behavior. 

In recent years, genomic and transcriptomic studies of medulloblastoma have been employed to identify key driver oncogenes and novel mutations [6,16,18,19,21,22,23,24], but prognostic significance of most of these molecular biomarkers is restricted to a specific subgroup of medulloblastoma (Table 1). Although Daoy and UW228 are commonly used in studies of medulloblastoma, very few proteomic studies related to Daoy and UW228 have been published [28,30], with almost no studies performed by integrated transcriptomic and proteomic analysis. Given the distinct developmental origins of the MB subtypes, driving the heterogeneity within and between the different subtypes, identification and validation of predictive and prognostic signature biomarkers for each subtype, with utility for diagnosis, management and follow-up of the disease may ultimately improve survival. 

We have previously demonstrated the value of using integrated proteomic and transcriptomic data to identify differentially expressed proteins with known cancer associations, as well as mapping protein interaction networks with significant perturbations in cancer [34]. In this report we provide the first detailed characterization of the Daoy and UW228 cell lines, two established primary medulloblastoma cell lines, by integrated transcriptomic and proteomic analysis. This approach identified a large number of common and unique genes and proteins in these cell lines, which may help provide greater insight into the differences in their biological behaviors. 

Differential expression of several proteins with known cancer association was observed between the two MB cell lines (Table 2). Notable among the proteins with differential expression between the two cell lines was the observed upregulation of several HSPs, including HSP90AA1 and HSP90AB1, in Daoy relative to UW228, is consistent with previous reports of their overexpression in tumor, relative to non-tumor tissues [53,54]. As the most abundant proteins in malignant cells, involved in stabilizing oncoproteins which regulate cancer growth and survival, increased expression of HSP90 proteins may play an important role in promoting aggressive cancer phenotypes, consistent with the reported invasive phenotype of Daoy cells [43].

Another interesting observation was the absence of L1CAM (CD171) in Daoy. Down regulation of L1CAM in SHH MB, relative to the other MB variants, has been previously reported [55], providing further evidence for the involvement of the SHH signaling pathway in Daoy. 

Gains of chromosomes 7 or 17 have been described in up to 60% of all medulloblastoma samples [56,57]. Interestingly, the set of genes amplified in Daoy, relative to UW228, included a number of genes located on chromosome 7 (*EGFR*, *LANCL2*) and 17 (*CRK*, *TP53*, *GRB2*, *TOP2A*, *JUP*). Gains in chromosome 7p11 [58] and chromosome 17p13, a region with a high incidence of loss of heterozygosity [59], have been reported in various cancers [60]. This may explain the high expression levels of genes located on both chromosomal regions in Daoy, which may correlate with its greater rate of cell division and migration and more invasive growth phenotype.

The observed differences in expression levels of key components of the SHH and WNT signaling pathways between Daoy and UW228 may further explain their reported different growth characteristics. Comparative transcriptomic analysis of the two signaling pathways (WNT and SHH) found high levels of genes with measurable transcripts for key components of both pathways; more than 80% of genes involved in these signaling pathways had RPKM values ≥0.3. Combining the proteomics and transcript data allowed discrimination of these pathways in the two cell lines. Within the WNT signaling pathway, upregulation of the PRK family is observed in Daoy, while the frizzled ligands as well as the “apoptosis sub-pathway” are predominant in UW228 (Figure 4A). Our work offers, for the first time, a detailed proteomic, transcriptomic and network analysis for two well-established primary medulloblastoma cell lines, Daoy and UW228. We identified a number of novel and well-established cancer associated proteins, together with unique overexpression of key SHH and WNT markers. Comparative expression analysis of key components of signaling pathways indicated SHH as the main signaling pathway in Daoy, and WNT as the primary signaling pathway involved in UW228.

In summary, the detailed description of the transcripts and proteins expressed in Daoy and UW228 and novel information on genes and proteins that are differentially expressed between these cell lines, may provide insights into the biology of these cell lines at the molecular level and help elucidate their MB subtype classification.

## Figures and Tables

**Table 1:** Patient/tumor characteristics of medulloblastoma subtypes and expression profiles of their associated genes.

Subtype	WNT	SHH	Group 3	Group 4
**Prevalence**	10%	30%	25%	35%
**Patients**	Common in children and adults, uncommon in infants.	Common in infants and adults, less common in children.	Common in infants and children, uncommon in adults.	Most common in children, less common in infants and adults.
	**Male = Female**	**Male = Female**	**Males > Females**	**Males > Females**
**Prognosis (5 year survival)**	Very good (95%)	Infants good others intermediate (75%)	Poor (50%)	Intermediate (75%)
**Histology**	Classic, rare large cell/anaplastic	Classic, large cell/anaplastic, desmoplastic, nodular	Classic, large cell anaplastic	Classic, large cell anaplastic
**Metastasis**	Rare (5%–10%)	Uncommon (15%–20%)	Very frequent (40%–45%)	Frequent (35%–40%)
**Genetic characteristics**	*CTNNB1* mutations	*PTCH1*, *SMO*, *SUFU* mutations; *GLI1*, *GLI2*, *MYCN* amplification	*MYC* amplification	*CDK6*, *MYCN* amplification
	WNT signaling			
	*MYC* over-expressed			

**Table 2:** Comparison of relative abundance of selected cancer-associated proteins (and their corresponding RNA levels) between UW228 and Daoy. Proteins involved in cell adhesion, actin organization, stress resistance, cell signaling and tumor suppression were found to be upregulated at both transcript and protein levels in UW228 cells relative to Daoy. Conversely, upregulated proteins in Daoy included several proteins involved in signal transduction, cell adhesion and DNA binding/repair. Majority of the amplified transcripts/upregulated proteins in Daoy are localized on chromosome 17, which is frequently gained in several malignancies, including medulloblastoma. (ND—not detected; #—Number).

Accession	Gene	Description	Chromosome	Daoy		UW228	
				RNA (RPKM)	# Unique Peptides	RNA (RPKM)	# Unique Peptides
**Overexpressed in Daoy, relative to UW228**							
P07900	**HSP90AA1**	Heat Shock Protein 90kDa Alpha Family Class A Member 1	14q32	421.65	18.00	167.03	7.00
P08238	**HSP90AB1**	Heat Shock Protein 90kDa Alpha Family Class B Member 1	6p21	775.45	19.00	362.14	9.00
P06748	**NPM1**	Nucleophosmin (Nucleolar Phosphoprotein B23, Numatrin)	5q35	668.25	5.33	239.64	3.00
Q15582	**TGFBI**	Transforming Growth Factor Beta Induced	5q31	3135.54	17.00	1249.52	7.00
Q00325	**DSP**	Desmoplakin	6q24	22.83	10.00	0.03	2.00
P24821	**TNC**	Tenascin C	9q33	241.59	8.00	37.95	1.00
P00533	**EGFR**	Epidermal Growth Factor Receptor	7p11	21.24	8.00	13.13	2.00
Q9NS86	**LANCL2**	LanC Lantibiotic Synthetase Component C-Like 2 (Bacterial)1	7p11	22.28	3.00	6.52	1.00
P62993	**GRB2**	Growth Factor Receptor-Bound Protein 2	17q24	44.9	5.00	30.56	1.00
P11388	**TOP2A**	Topoisomerase (DNA) II Alpha	17q21	129.62	8.00	45.76	0.00
P14923	**JUP**	Junction Plakoglobin	17q21	56.97	7.00	19.84	1.00
P46108	**CRK**	V-Crk Avian Sarcoma Virus CT10 Oncogene Homolog	17p13	50.20	8.00	17.16	1.00
P04637	**TP53**	Tumor Protein P53	17p13	235.10	5.00	36.74	ND
P62826	**RAN**	RAN, Member RAS Oncogene Family	12q24	479.63	6.00	240.29	3.00
P43246	**MSH2**	MutS Homolog 2	2p21	18.07	6.00	10.87	ND
P52701	**MSH6**	MutS Homolog 6	2p16	25.35	8.00	19.98	1.00
**Overexpressed in UW228, relative to Daoy**							
P02511	**CRYAB**	Crystallin Alpha B	11q23	0.74	ND	438.60	4.67
Q14847	**LASP1**	LIM And SH3 Protein 1	17q12	59.55	10.00	124.73	18.00
Q15063	**POSTN**	Periostin	13q13	6.53	ND	1023.51	6.00
P43121	**MCAM**	Melanoma Cell Adhesion Molecule	11q23	36.97	ND	61.89	10.33
P32004	**L1CAM**	L1 Cell Adhesion Molecule	Xq28	1.15	ND	324.27	10.33
P46939	**UTRN**	Utrophin	6q24	3.7	1.00	9.86	12.00
Q07954	**LRP1**	Low-density lipoprotein receptor-related protein 1	12q13	6.73	2.00	79.98	13.67
P80723	**BASP1**	Brain Abundant Membrane Attached Signal Protein 1	5p15	63.7	5.00	270.37	9.00
P98082	**DAB2**	DAB2, Clathrin Adaptor Protein	5p13	7.8	ND	68.7	2.00
Q6UVK1	**CSPG4**	Chondroitin Sulfate Proteoglycan 4	15q24	6.8	3.00	17.8	10.00
Q01995	**TAGLN**	Transgelin	11q23	27.27	8.00	353.95	12.00

## Supplementary Materials

The following are available online at www.mdpi.com/2227-7382/5/1/5/s1. Table S1: List of non-redundant proteins detected in Daoy and UW228, with 2 or more unique peptides, Table S2: List of proteins overexpressed in Daoy, relative to UW228, Table S3: List of proteins overexpressed in UW228, relative to Daoy, Table S4: RNA-Seq data for Daoy and UW228.

## Abbreviations

The abbreviations used are:

MB	Medulloblastoma;
LC	Liquid chromatography
MS/MS	Tandem mass spectrometry
RNASeq	RNA sequencing
SHH	Sonic hedgehog
D/N	Desmoplastic/nodular
MBEN	Medulloblastoma with extensive nodularity
CTNNB1	Beta-catenin
DDX3X	DEAD-box RNA helicase gene
SMARCA4	SWI/SNF related, matrix associated, actin dependent regulator of chromatin, subfamily A, member 4
TP53	Tumor suppressor P53
KMT2D	Lysine (K)-specific methyltransferase 2D
PTCH1	Patched 1
GLI1/GLI2	GLI family zinc finger 1 or 2
*KDM6A*	Lysine (K)-Specific Demethylase 6A
CDK6	Cyclin-Dependent Kinase 6
NPM1	Neuplasmin
EZR	Ezrin
SET	SET nuclear oncogene
TGFBI	Transforming growth factor, beta induced
MCAM	Melanoma cell adhesion molecule
POSTN	Periostin, osteoblast specific factor
SUFU	Suppressor of fused homolog
SMO	Smoothened, frizzled class receptor
CRK	V-Crk avian sarcoma virus CT10 oncogene homolog
LASP1	LIM And SH3 protein 1
PCNA	Proliferating cell nuclear antigen
JUP	Junction plakoglobin
CRYAB	Crystallin Alpha B
